# Timing in turn-taking and its implications for processing models of language

**DOI:** 10.3389/fpsyg.2015.00731

**Published:** 2015-06-12

**Authors:** Stephen C. Levinson, Francisco Torreira

**Affiliations:** ^1^Language and Cognition Department, Max Planck Institute for PsycholinguisticsNijmegen, Netherlands; ^2^Donders Institute for Brain, Cognition and Behaviour, Radboud UniversityNijmegen, Netherlands

**Keywords:** turn-taking, conversation, language processing, language production, language comprehension

## Abstract

The core niche for language use is in verbal interaction, involving the rapid exchange of turns at talking. This paper reviews the extensive literature about this system, adding new statistical analyses of behavioral data where they have been missing, demonstrating that turn-taking has the systematic properties originally noted by Sacks et al. (1974; hereafter SSJ). This system poses some significant puzzles for current theories of language processing: the gaps between turns are short (of the order of 200 ms), but the latencies involved in language production are much longer (over 600 ms). This seems to imply that participants in conversation must predict (or ‘project’ as SSJ have it) the end of the current speaker’s turn in order to prepare their response in advance. This in turn implies some overlap between production and comprehension despite their use of common processing resources. Collecting together what is known behaviorally and experimentally about the system, the space for systematic explanations of language processing for conversation can be significantly narrowed, and we sketch some first model of the mental processes involved for the participant preparing to speak next.

## 1. Introduction: Why Turn-Taking in Conversation is Important for the Psychology of Language

One of the most distinctive ethological properties of humans is that they spend considerable hours in the day in a close (often face-to-face) position with others, exchanging short bursts of sound in a human-specific communication pattern: extrapolating from Mehl et al. (2007), we may each produce about 1200 of these bursts a day, for a total of 2–3 h of speech. The bursts tend to involve a phrasal or clausal unit, but can be longer or shorter. At the end of such bursts, a speaker stops, and another takes a turn. This is the prime ecological niche for language, the context in which language is learned (see Section 6.1 below), in which the cultural forms of language have evolved, and where the bulk of language usage happens.

This core form of language use poses a central puzzle for psycholinguistics (see Section 6), which has largely ignored this context, instead examining details of the processes of language production or comprehension separately in laboratory contexts. Yet this prime use of language involves rapid switching between comprehension and production at a rate implying that these processes must sometimes overlap. Decades of experimentation have shown that the language production system has latencies of around 600 ms and up for encoding a new word (reviewed in Section 6.3) but the gaps between turns average around 200 ms (see Section 5). This would seem to imply that participants planning to respond are already encoding their responses while the incoming turn from the other speaker is still unfinished. This in turn implies potentially long-range prediction in comprehension. A sketch model of the interleaving of comprehension and production processes is presented in Section 7.

To appreciate the full nature of this puzzle, it is essential to review what we know about the turn-taking system and its temporal properties. In Section 2, we review the foundational Sacks et al. (1974; henceforth SSJ) model of turn-taking, considering alternative proposals in Sections 3 and 4. The model proposes extensive prediction (or ‘projection’) of turn-ends, and an expectation of swift response. The systematicity of turn-taking and its temporal patterning are borne out by extensive corpus analyses (Section 5). We then turn to the psycholinguistic literature (Section 6), noting that sensitivity to turn-end cues is already shown early in child development. We point out that there is considerable evidence for predictive language comprehension, and for long latencies in language production, so that the central psycholinguistic puzzle (Section 6.5) posed by turn-taking seems to be resolved by predicting what the other interlocutor is going to say. Some direct recent investigations seem to bear this out (Section 6.4), although experimentation in this field is in its infancy. In Section 7 we take stock of the recent findings, and sketch a processing model addressing some of the processing puzzles involved.

## 2. Turn-Taking as a System: Research from Conversation Analysis

Sacks et al. (1974; SSJ) initiated the modern literature on conversational turn-taking by outlining how this behavior constitutes a system of social interaction with specific properties. It is not organized in advance (by say an order of speaking, or set units to be uttered), but is highly flexible, allowing for longer units when so mutually arranged, and organizing an indeterminate number of participants into a single conversation. The authors note that “overwhelmingly one speaker talks at a time. Occurrences of more than one speaker at a time are common but brief […] Transitions (from one turn to the next) with no gap and no overlap are common, and together with slight gaps and slight overlaps make up the majority of transitions” (Sacks et al., 1974, p. 700). Obviously, such turn-taking behavior contrasts with the absence of turn-taking in cheering, heckling, laughing, etc. That things could be otherwise in the speech domain is shown by the contrasting speech exchange systems we also use, as in lectures where questions come at the end, or in a press conference where questions come from many parties but are answered by one, contrasting with a classroom where questions may come from the teacher alone, and may be answered by many volunteers. The importance of the conversational system is that, unlike the others, it appears to be the default mode of language use, as shown by its operation in the context of language learning, and among friends and family. As far as we know, it operates in a strongly universal way (cf. Stivers et al., 2009, 2010), while the other speech exchange systems are mostly culture-specific.

Sacks et al. (1974) argued that conversation is an elemental piece of social organization, regulated by social norms that prescribe one speaker at a time but allow open participation. The model they suggested consists of turn units and rules that operate over those units. The units they suggested are variable sizes of syntactic units, whose functions as full turns can be indicated prosodically. The end of such a unit constitutes a ‘transition relevance place’ or TRP. The rules specify:

(1) If the current speaker C selects the next speaker N, then C must stop, and N should start. (‘Selection’ could involve address terms, gaze, or in the case of dyadic conversation defaults to the other.)
(2) If C does not select N, than any participant can self-select, first starter gaining rights to that next unit.
(3) If no other party self-selects, C may continue.

These rules then recursively apply at each TRP.

These rules predict that intra-speaker silent gaps (generated by rule 3) will be longer than inter-speaker ones, a fact shown to be correct on large samples of conversation [ten Bosch et al., 2005 report gaps between continuations by the same speaker to be about 140 ms (c. 25%) longer than the average gap in turn transitions between different speakers]. It has also been suggested that on this basis a turn-taking ‘beat’ or ‘clock’ (with a period between 80 and 180 ms) can be discerned, suggesting a model of coupled oscillators that allow participants to synch (Wilson and Zimmerman, 1986; Wilson and Wilson, 2005).

It was evident to Sacks et al. (1974) that the model had consequences for language processing. They noted that, given that interlocutors may be addressed at any point, the system enforces obligate listening. More importantly, they noted that the speed of speaker transition would require ‘projection’ (prediction) of the end of the incoming turn, and production processes would have to begin before the end of the incoming turn, in part because turn beginnings have to be designed to facilitate that very projection (Sacks et al., 1974, 719; Levinson, 2013). Later corpus studies have established, as we shall see (see Section 4), that the great proportion of turn transitions fall between -100 and 500 ms, that is, between a short stretch of overlap to a gap with a duration equivalent to one to three syllables.

There is a great deal of later work in conversation analysis (CA) that has contributed to our understanding of this system (see Clayman, 2013; Drew, 2013; Hayashi, 2013 for overviews). It is important to appreciate that not all overlapping of turns can be understood as behavior that violates the rules above – some authors (see Section 4) have seen the frequency of overlap as undercutting the Sacks et al. (1974) model. Sacks et al. (1974) claimed that overlaps are common, but usually very short, and often accounted for by little additions to the first turn like address forms or tags [as in (1)], or by misanalyses of when the turn is coming to an end [as in (2) where ‘biscuits’ was projected as the turn-end but it was followed by ‘and cheese’; overlap indicated with square brackets]:

(1) Sacks et al. (1974, p. 707)
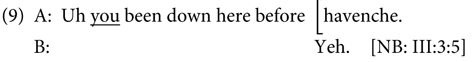

(2) Jefferson (1984, p. 15)
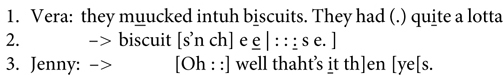

Note especially that some overlaps – namely competing (more or less simultaneous) first starts – are expectable by the rules above (as when two people start simultaneously by rule 2, or a participant operating rule 2 is a bit slow and overlaps with the current speaker continuing by rule 3). In these cases one or the other of the speakers normally drops out (impressionistic gap duration in seconds between brackets):

(3) Hayashi, 2013, p. 176 (from Auto Discussion)
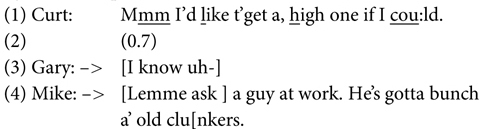

When there is competition to maintain the floor in these and other cases, this is often negotiated on a syllable by syllable basis, with e.g., deceleration, increase of intensity, and repeated syllables or words, until one speaker drops out (Schegloff, 2000).

Just as different kinds of overlap can be discerned, so can different kinds of absence of speech, differentiating between pauses (e.g., between units by the same speaker), gaps (between speakers), silences (meaningful absence of speech, e.g., after a question), and lapses (where no-one has self-selected to speak). It has been suggested (citations below) that participants are very sensitive to timing, so that an excessively long gap after a question, for instance, may be taken to indicate that the recipient has some kind of problem with it, for example finding it difficult to answer in the affirmative, or has uncertainty about the response. In the following a telephone caller takes gap of around 2 s to indicate the answer ‘no,’ which he himself then pre-emptively provides:

(4) Levinson, 1983, p. 320
  C: So I was wondering would you be in your office on Monday (.) by any chance?
  (2.0 s)
  C: Probably not.

A considerable body of work has gone into understanding the role of extended gaps or silences in ‘dispreferred’ responses (responses not in line with the suggested action in the prior turn; see Pomerantz and Heritage, 2013 for review). Corpus analysis shows that gaps of 700 ms or more are associated with dispreferred actions, and that gaps longer than the norm (>300 ms) decrease the likelihood of an unqualified acceptance, and increase the likelihood that a response, be it acceptance or rejection, will have a dispreferred turn format (e.g., *Yes, but…* in the cases of acceptances; Kendrick and Torreira, 2015). Experimental work also shows that gaps of 600 ms or longer generate inferences of this unwelcome kind (Roberts et al., 2011).

The CA approach to turn-taking raises two major issues. The first is what exactly counts as a turn, and how participants can recognize such a unit as complete. The problem is that just about any word or phrase may in context constitute a turn, while syntactic units can be nested or conjoined indefinitely. Regarding this issue, Sacks et al. (1974, p. 721) note that “some understanding of sound production (i.e., phonology, intonation, etc.) is also very important to turn-taking organization.“ Thus in the following (drawn from the discussion in Clayman, 2013, p. 155), the terminal intonation contours do not occur till the end of the turns, and two turns each composed of three possibly complete syntactic units (divided by §) occur uninterrupted (note the whole is recognized by the recipient as a story under way, hence the continuers, which are themselves possibly elicited by rising intonation marked with ‘?’):

(5) Ford and Thompson (1996, p. 151)
  K: Vera (.) was talking § on the phone § to her mom?
(6) C: mm hm
  K: And uh she got off § the phone § and she was
  incredibly upset?
  C: Mm hm.

In addition to syntactic and prosodic completeness, pragmatic completeness may be required to terminate a turn (Ford and Thompson, 1996; Levinson, 2013). Clearly a responsive action following the first part of a pair of actions like questions and answers, offers and acceptances, requests and compliances can be inspected for pragmatic efficacy; elsewhere the larger role in a sequence of speech acts may need to be satisfied.

The second major issue is ‘projection’ or predictive language understanding. Sacks et al. (1974) thought it clear that the turn-taking system can only work if there is extensive prediction in comprehension, so that recipients can use the unfolding turn to project an overall syntactic and prosodic envelope which would allow them to foresee when and how a turn would come to an end (see Clayman, 2013 for a review). It is not at all clear how this works, given the flexibility and extendibility of most syntactic units. Still, interesting insights are provided by such phenomena as turn-completion by the other, studied in depth by Lerner (1991, 2002; see also Hayashi, 2013). A typical example is where a bi-clausal structure is begun by speaker A, and the second clause completed by speaker B as below. Clearly an *If..then..* or *Whenever…, X…* structure projects a second downstream clause.

(7) Lerner (1991, p. 445)
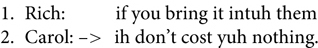

Such cases do not alone show that recipients accurately predict the content of the second clause (indeed sometimes a jokey exploitation of the structure may appear). But sometimes exactly the same words do occur in overlap:

(8) Lerner (1991, p. 239)
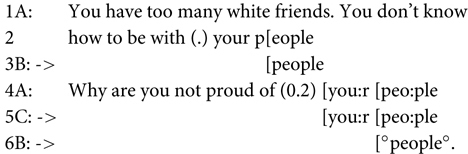

Such intrusions into others’ turns are rare, and can act as demonstrations of understanding, occurring more routinely if speaker A is obviously engaged in a word-search and speaker B can provide the item. Cases like these demonstrate that extensive projection is possible, and psycholinguistic evidence supports this (Predictive Language Comprehension).

## 3. An Alternative Signaling Approach

The term ‘turn-taking’ was independently suggested by Yngve (1970) and Duncan (1972). Contemporaneous with the approach by Sacks et al. (1974), Duncan (1972, 1974) proposed, using videotapes of dialogs, a set of turn-taking signals. The main set are turn-handing-over signals, and consists of half a dozen cues: prosodic (type of final intonation, final syllable duration, final drop in pitch, or loudness), gestural (end of a gesture), and lexical/syntactic (tag, clause end, etc.). A second proposed signal is turn-maintaining and consists of a final mid-tone, continuing gesture or a gaze switch away. Turns followed by speaker change were found to nearly always occur with one or more turn-ending cues. On this basis, Duncan advanced a model where the turn-taking system is entirely under the control of the current speaker. This contrasts with the CA model, where speaker transition is contingently achieved by one speaker coming to the end of a unit and another starting (e.g., by self-selection). In addition, in the CA model there are no context-free signals: e.g., in English, a final mid tone usually marks turn-holding, but in specific contexts it may indicate turn-yielding (as when the conjunction *or* is appended to polar questions, e.g., *Are you leaving, or…?*); thus turn-taking can only be achieved on some much more global understanding of the incoming turn.

Although the signaling view is largely superseded, the research drew attention to (a) the importance of visual cues, and (b) the coincidence of turn transitions with a number of features of turn construction, prosody, gesture, etc. Kendon (1967) had earlier described different patterns of gaze between speakers (who alternately look away and look to addressees) and addressees (who gaze longer at the speaker). Goodwin (1980) later proposed a rule that sometime during the course of a turn a speaker should glance at the addressee, expecting to find a gazing addressee whenever he or she looks. The idea that speaker gaze when returning to addressee could function as a turn-yielding cue is, however, not easy to substantiate; More recently, Rossano (2013) has suggested this is because gaze is actually oriented to larger units of conversation (sequences), which it may serve to open and close.

## 4. Challenges to the Standard Model

Recently an alternative view to the Sacks et al. (1974) account was advanced by Heldner and Edlund (2010), who argue that turn-taking does not have all of the systematic properties described by Sacks et al. (1974). First, they find fault with the claim that speakers aim at no gap and no overlap. Actual zero gaps (under 10 ms) represent less than 1% of transitions and overlaps average 40% of transitions in their corpora. “From these observations, we conclude that the target with respect to timing of turn-taking cannot be one-speaker-at-a-time and no-gap–no-overlap, and furthermore that precision timing in turn-taking can neither be used in arguments in favor of projection, nor against reaction as models of timing in turn-taking” (Heldner and Edlund, 2010, p. 567). We believe these conclusions are misguided, and spell out the reasons here.

First, a target of 10 ms precision may not be realistic of human performance. Voiceless stops in English average between 60 and 80 ms (Crystal and House, 1988; Byrd, 1993), and at the end of a turn will be hard to distinguish from the beginning of the gap. Perceptual “no gap” was always estimated by conversation analysts to be of the order of 150–250 ms (i.e., close to the speaker transition mode; Schegloff, 2000). Heldner (2011) himself has gone on to show most usefully that a gap or overlap under 120 ms is not perceived as gap or overlap, respectively. It is interesting to compare the tolerable degree of lag in cross-modal matching as in the McGurk effect: an auditory signal following a visual one by up to 180 ms will still seem to be synchronized (Munhall et al., 1996). The majority (51–55%) of all turn transitions across corpora take place in under 200 ms (Heldner and Edlund, 2010, p. 563).

Second, as explained above, overlaps are of different kinds, some (e.g., continuers like *hmhm*, or minimal terminal overlaps) not being heard as intrusions on the turn, and others (like competing first starts) being specifically expectable. Below we provide a quantitative study of overlap (Overlap), which shows that overlaps tend to be minimal in size and occupy less than 5% of the speech stream.

Meanwhile, the argument that there is no target to avoid overlap seems unlikely. Qualitative analysis shows, as mentioned, that when overlap occurs, one speaker tends to rapidly drop out [as in example (3) above] so that the bulk of overlaps are of short duration. ‘Interruption’ is a sanctionable breach of social mores, as every child learns. The systematic properties of all the corpora that have been studied would be entirely different if overlap was not avoided.

On the basis of their dismissal of the no-gap–no-overlap target, Heldner and Edlund (2010, p. 566) go on to attack further aspects of the standard model: “Thus, the no-gap–no-overlap principle (Sacks et al., 1974) can neither be used as a part of an argument in favor of projection nor against reaction simply because the no-gap–no-overlap cases hardly ever occur in real speaker change data. Importantly, this means that a principal motivation for projection in turn-taking is invalid.” This attack on projection as a central element of the model will prove misplaced when we turn to consider the psycholinguistic evidence below (in fact Heldner and Edlund, 2010, p. 566 later concede that projection of content may be responsible for overlaps and short gaps).

The central plank of the dismissal of projection is that turn-taking is often not as rapid as has been claimed. Heldner and Edlund (2010, p. 563) note:

“The cumulative distribution above the 200 ms threshold was also of interest, as it represented the cases where reaction to cessation of speech might be relevant given published minimal reaction times for spoken utterances (Fry, 1975; Izdebski and Shipp, 1978; Shipp et al., 1984). The distribution above this threshold represented 41–45% of all between-speaker intervals. These cases were thus potentially long enough to be reactions to the cessation of speech, or even more so to some prosodic information just before the silence.”

There are two separate proposals here. The first is that for gaps longer than 200 ms, participants might simply react to silence. This threshold is implausible. First, silence will only become recognizable as silence after c. 200 ms (after all the duration of voiceless stop consonants ranges up to 180 ms; cf. Heldner and Edlund, 2010), at which point it will still take a further minimally 200 ms to react (so 400 ms in total). That minimal reaction is for a prepared vowel (Fry, 1975), and any more complex response will increase according to Hick’s Law (see below); a choice between one of two prepared responses takes 350 ms for example. We now have, say, 550 ms from actual cessation of speech till beginning of a minimal response, and as Heldner and Edlund (2010) note 70–82% of responses are within 500 ms. Thus reaction to silence, although certainly possible in a minority of cases, would not seem to play a major role in the organization of turn-taking (see Riest et al., 2015).

The second proposal is that there is the possibility of reaction to “some prosodic information just before the silence.” Here there is less room for disagreement; CA practitioners and associated phoneticians have themselves emphasized the role of turn-final intonational and segmental cues (see Walker, 2013 for a review). Duncan drew attention to turn-keeping intonation cues and lengthened (‘drawled’) syllables. Critical here are two factors: (a) it must be shown not only (as Duncan did) that there are available prosodic/phonetic features of turn-ends, but also that participants actually use them, (b) the location of the features with respect to the turn end is important (e.g., sentence accents in English sometimes occur well before turn ends, in which case talk of projection suits better than talk of reaction to terminal cues, cf. Wells and Macfarlane, 1998). Bögels and Torreira (in press) provide experimental evidence that listeners do use turn-final prosodic information (located in the last syllable of the utterance) to identify turn ends in Dutch questions with final rising intonation. Further research should investigate other linguistic contexts.

Another notion that has some currency is that turn-taking could be driven by coupled oscillators (Wilson and Wilson, 2005). Coupled oscillators have been shown to play a role in coordination in the animal world, e.g., in the synchronization of fire-fly flashing where an individual’s flashes reset the neighboring fireflies’ oscillators, so gradually converging on a single beat. However, it is well known that human synchronization does not primarily work in this way, but rather by means of temporal estimation, which is easily shown by demonstrating that humans can tap together without waiting to hear the others’ taps (Buck and Buck, 1976). Moreover, given the highly variable lengths of turns, nothing like the firefly mechanism can work in conversation. Indeed, human coordination in general relies on simulating the other’s task, thus on high-level cognition (Sebanz and Knoblich, 2008). There is, however, room for a low level metronome, as it were, and Wilson and Wilson (2005) suggest that readiness to speak is governed by the syllable, so that participant A’s beginning of a syllable tends to coincide with B’s least readiness to speak, while the end of the syllable coincides with B’s increased readiness. There is indeed some evidence for entrainment or accommodation of the gap size between specific dyads, but there is no such effect on intra-turn pauses (ten Bosch et al., 2005) suggesting that turn-transition timing is rather unconnected to other temporal properties of speaking, although more research is required here.

Careful observers have convinced themselves that such a ‘beat’ is set up in English conversation by stress-timing, such that interlocutors producing unmarked actions with their turns tend to come in ‘on the beat’ (Couper-Kuhlen, 2009). However, the perceived rhythm of speech does not appear to have direct acoustic correlates, and to date we are unable to objectively confirm these observations (note too that languages differ in their rhythmic properties). Interestingly, recent corpus measurements show that, rather than the entrainment of a conversational beat, there is a reverse correlation of speaker A’s speech rate and speaker B’s response timing, perhaps because B has less time to plan her message as A’s speech rate increases, and vice versa (Roberts et al., 2015).

## 5. Statistical Studies of Corpora

The statistical study of turn-taking began early, prompted by developments in telephony, with a special interest in the speed of turn-transition (e.g., Norwine and Murphy, 1938). It has become standard to represent overlaps and gaps on a single time scale [sometimes called ‘the floor transfer offset’ (FTO)] in which positive values correspond to gaps, and negative values represent overlap. **Table 1** summarizes average values of FTOs in ten languages as reported in four studies (caveat: codings and methods differ somewhat in these studies). Note that although mean values vary, they do so in narrow window, roughly a quarter of a second either side of the cross-linguistic mean, and that the factors affecting response times are uniform across cultures (Stivers et al., 2009). In the following two sections, we look in more detail at the distribution of gaps and overlaps.

**Table 1:** Average floor transfer offsets (FTOs) in ten different languages as reported by four different studies.

Language	Average FTO (ms)	Source
English	410	Norwine and Murphy (1938)^∗^
English	480	Sellen (1995)^∗^
English	460	Sellen (1995)
Dutch	-78	De Ruiter et al. (2006)^∗^
Japanese	7	Stivers et al. (2009)
Tzeltal	67	Stivers et al. (2009)
Y’elî-Dnye	71	Stivers et al. (2009)
Dutch	108	Stivers et al. (2009)
Korean	182	Stivers et al. (2009)
English	236	Stivers et al. (2009)
Italian	309	Stivers et al. (2009)
Lao	419	Stivers et al. (2009)
Danish	468	Stivers et al. (2009)
ǂĀkhoe Haiǁom	423	Stivers et al. (2009)

^*^No eye-contact between conversation participants.

### 5.1. Distribution of Gaps

About half a century ago, Brady (1968) reported average gap durations of 345–456 ms and medians from 264 to 347 ms (depending on the threshold used in the automatic detection of speech) in a corpus of sixteen telephone calls between friends in the USA. Task-oriented interaction shows surprisingly similar patterns [e.g., Verbmobil – a travel scheduling task by telephone, has geometric means of 380 ms (English), 363 ms (German), 389 ms (Japanese); Weilhammer and Rabold, 2003]. In a wide review, Heldner and Edlund (2010) looked at three different corpora, automatically processing two of them for speaker transitions: a Dutch dialog corpus, and English and Swedish Map Tasks (where interlocutors must adjust their positions on slightly mismatching maps). The first two corpora included both face-to-face and non-face-to-face interaction. Heldner and Edlund (2010) found closely matching patterns across corpora, with combined scale (FTO) modes for speaker transition at c. 200 ms (i.e., a short gap) and c. 60% of transitions being gaps, 40% overlaps (including any overlap of greater than 10 ms; the modal overlap is less than 50 ms in the Spoken Dutch Corpus). Around 41–45% of gaps were longer than 200 ms, and between 70 and 82% of all transitions were shorter than 500 ms.

These quantitative approaches generalize over all kinds of speech acts and responses. But there is also growing work focused specifically on question–answer timings. Question–answer sequences are an interesting context to examine, because questions make a floor transfer relevant, whereas in other contexts a floor transfer between speakers is often optional. Stivers et al. (2009) looked at 10 languages from around the world, including smaller, unwritten languages, and found rather fast transitions in polar question contexts, with means between 7 and 468 ms, and modes from 0 to 200 ms. The coding of this sample was from videotape and included early visual responses (e.g., nods) and audible pre-utterance inbreaths. The general finding was that although languages differ, e.g., in their degree of use of visual modality or mean response times, the factors that speeded or slowed response times (e.g., gaze, agreement) were shared. Heldner (2011) shows that estimates of the percentage of perceived overlaps and gaps in this sample match closely other quantitative samples.

The intensive study of turn-taking under different conditions is still in its infancy. We know that responses to Wh-questions are slower than polar (yes–no) questions cross-linguistically (unpublished data from the Stivers et al., 2009 study), presumably because of the greater cognitive complexity of response involved. Longer answers can also be shown to take more preparation, reflected in both reaction times, and breathing preparation (Torreira et al., 2015). Complexity of response has also been shown to influence timings in children’s responses (Casillas, 2014). We also know that individuals tend to accommodate to the gap-length of others, so that when changing conversational partners, individuals’ response times change to match their new interlocutors (ten Bosch et al., 2004, 2005). And intriguingly, transition speeds are higher on the phone than face-to-face (Levinson, 1983; ten Bosch et al., 2005).

#### 5.2. Overlap

In contrast to gaps, the study of overlap in corpora has provided only gross facts. As mentioned, Heldner and Edlund (2010) report c. 40% of speaker-transitions involving overlaps (including any overlap of greater than 10 ms). Their histogram makes clear that the modal overlap is less than 50 ms in the Spoken Dutch Corpus, with a mean -610 ms, and median -470 ms. ten Bosch et al. (2005) report that the proportion of overlaps increases from 44% in face-to-face conversation to 52% in telephone conversation, with males more likely to overlap their interlocutor than females, but looking just at the transition from speaker A to speaker B, 80% of transitions are gaps and 20% partial overlaps in face-to-face conversation (the corresponding figures for telephony are 73 and 27%).

Because of the lack of detailed statistical analysis of overlaps in corpora, we have undertaken a new analysis of overlaps in the Switchboard Corpus of English telephone conversations (Godfrey et al., 1992). We address the following questions:

(1) In running speech, how common is overlap (i.e., simultaneous talk by more than one party at a time) compared to talk by one party alone?
(2) In floor transfers, how common are overlaps compared to gaps?
(3) What is the distribution of overlap duration, and where do overlaps tend to start relative to the interlocutor’s turn?
(4) What is the distribution of different overlap types (cf. Jefferson, 1986)?

#### 5.2.1. Method

**FIGURE 1:**
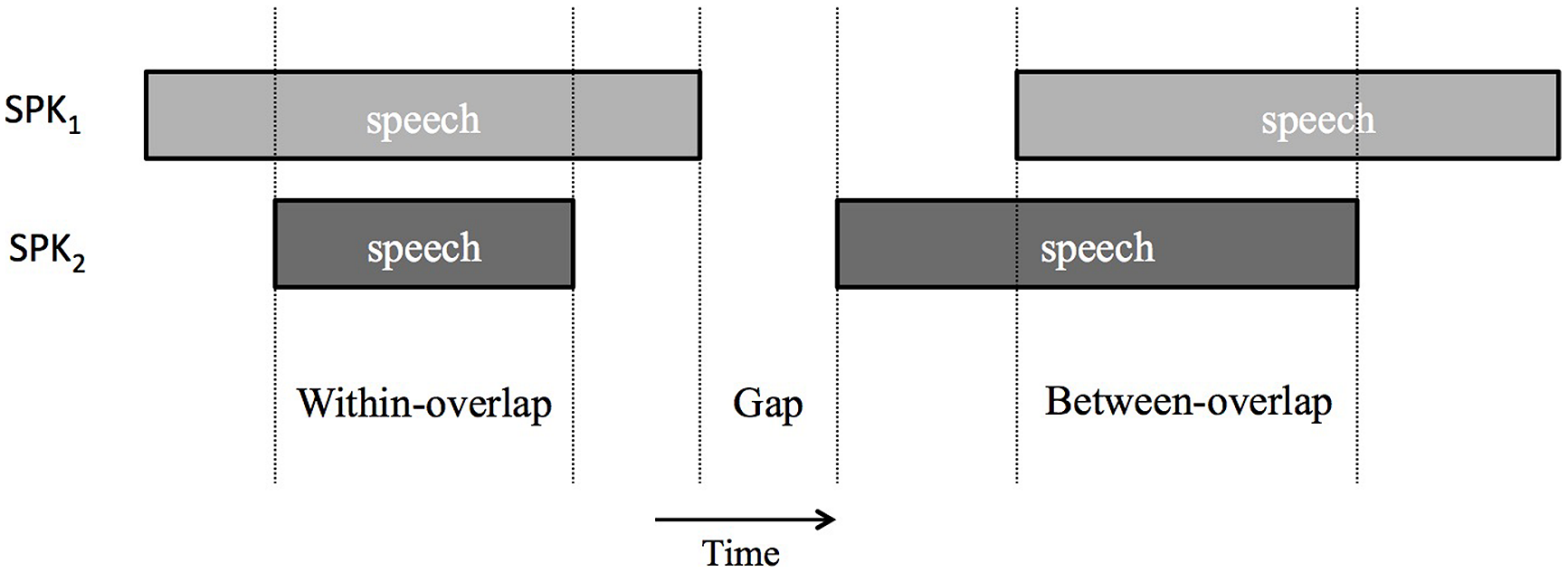
**Illustration of gaps, within-overlaps, and between-overlaps for two speakers (SPK_1_ and SPK_2_) in our classification scheme following Heldner and Edlund (2010)**.

We analyzed a subset of 348 conversations (totaling around 38 h of dyadic conversation) that were free of timing errors, and with annotations included in the NXT-Switchboard Corpus release (Calhoun et al., 2010). To estimate the occurrence of overlaps in this dataset, we used the operationalization scheme in Heldner and Edlund (2010). First, based on the phonetic segmentation of the corpus, we divided each speaker’s signal into interpausal units (IPUs) delimited by silent intervals of 180 ms or more. The 50,510 IPUs had an average duration of 1680 ms, and a median duration of 1227 ms. Second, we defined *gaps*, *pauses*, *between-overlaps,* and *within-overlaps* as follows. Gaps (*n* = 14648) corresponded to portions of the stereo signal that contained silence in each speaker’s channel, and that involved a floor transfer between the two speakers. Between-overlaps (*n* = 6524) were floor transfers that occurred without a silent gap between the speakers, whereas within-overlaps (*n* = 3343) were parts of the signal with overlapping inter-pausal units that did not result in an effective floor transfer. **Figure 1** below illustrates the operationalization of gaps, between-overlaps and within-overlaps.

#### 5.2.2. Findings

The recordings were divided as follows: 77% of the signal corresponded to speech by one speaker only, 19.2% to silence (i.e., either pauses within a speaker’s turn or gaps as defined above), and only 3.8% to simultaneous speech by both speakers (either between-overlaps or within-overlaps). If we exclude silent parts, 95.3% of the speech signal corresponded to speech by one speaker. This seems to fit well with Sacks and colleagues’ observation that “overwhelmingly, one party speaks at a time” (Sacks et al., 1974, p. 700).

**FIGURE 2:**
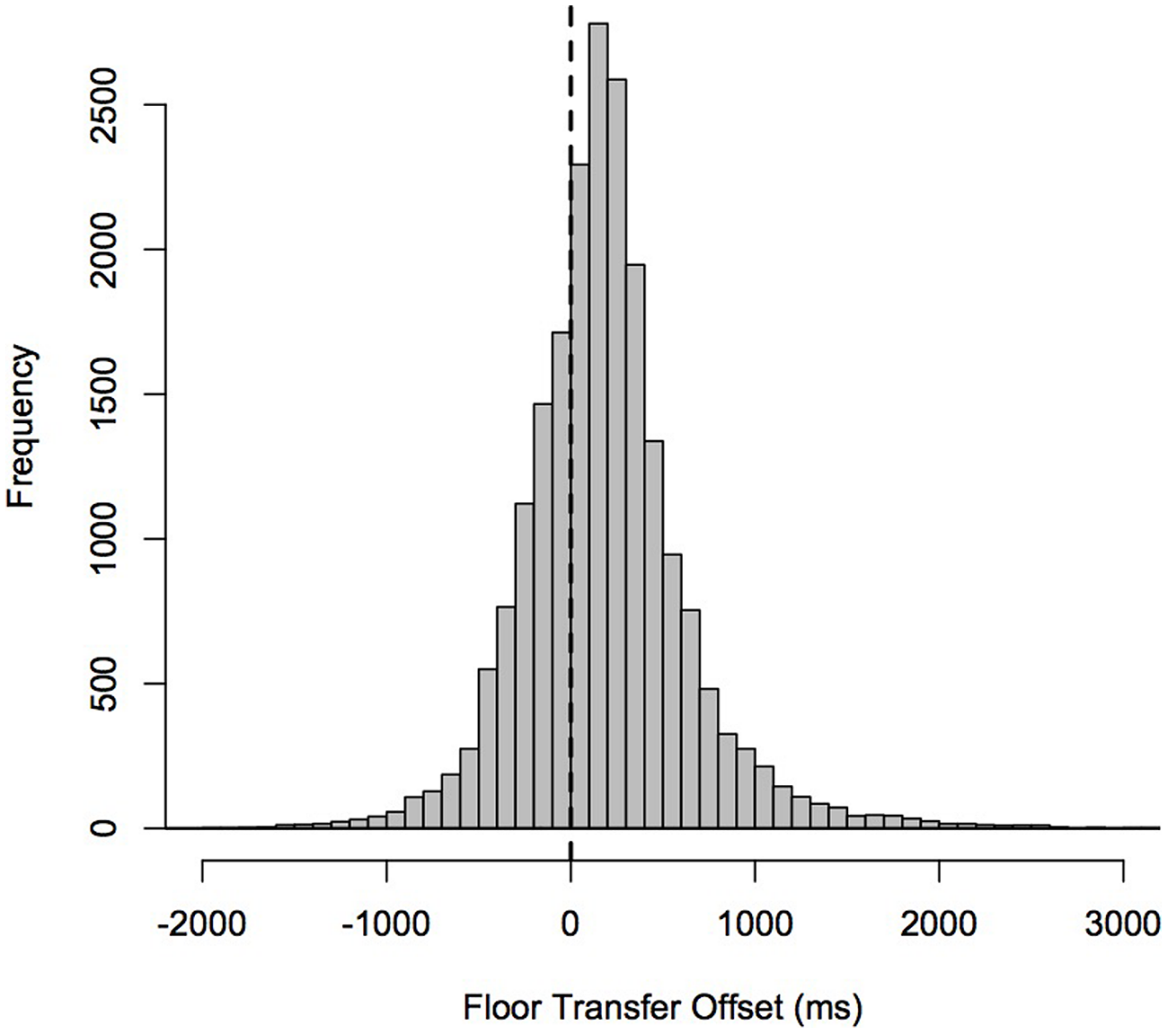
**Histogram of floor transfer offsets (FTOs) in the Switchboard Corpus (Godfrey et al., 1992; Calhoun et al., 2010, see Section 5.2.1 for details).** Each bin has a size of 100 ms.

With regard to how common overlaps are in terms of proportion of turn-transitions, **Figure 2** shows the distribution of the duration of gaps and between-overlaps combined together as FTOs (i.e., with positive values for gaps and negative values for between-overlaps). Between-overlaps (negative FTOs) represented 30.1% of all floor transfers. As for the duration of overlaps, and their location within the interlocutor’s turn, we observed that between-overlaps exhibited a distribution highly skewed to the left, with an estimated modal duration of 96 ms, a median of 205 ms, a mean of 275 ms, and with 75% of the data with values below 374 ms. Within-overlaps tended to start close to the beginning of the utterances that they overlapped, with a modal offset of 350 ms, a median of 389 ms, a mean of 447 ms, and 75% of the data exhibiting offsets below 532 ms. Their duration exhibited a distribution highly skewed to the right, with an estimated modal duration of 350 ms, a median of 389 ms, a mean of 447 ms, and 75% of the data with values below 532 ms. The duration of within-overlaps is thus usually shorter than that of two syllables. This appears to fit well with Sacks et al.’s (1974) observation that “occurrences of more than one speaker at a time are common, but brief.”

We now examine the distribution of different types of overlaps. A prediction made by the Sacks et al. (1974) model is that most overlaps should be occasioned by a number of circumstances emerging from the application of its rules. For instance: (i) Overlaps often arise when unforeseen additions to the first speaker’s turn after a transition relevance place (e.g., during increments or tags); (ii) They may occur after a silence when two speakers may self-select and launch articulation without realizing that another party is doing the same thing (cf. ‘blind spot’ cases, Jefferson, 1986); (iii) They may frequently arise in cases involving backchannels signaling feedback to the main speaker (e.g., *yeah*, *right*) and other minimal utterances that do not constitute an attempt to take the floor. The Sacks et al. (1974) model also predicts signs of overlap avoidance when it occurs, for instance by speakers’ abandoning their turns without reaching a point of turn completion. Another sign of speakers’ special orientation to overlapping talk is that they may engage in competition for the floor, for instance by repeating syllables or words, often with increased intensity and pitch levels (Schegloff, 2000).

To estimate the prevalence of such possible causal contexts for overlap, in a separate analysis we randomly sampled 100 between-overlaps and 100 within-overlaps from our data, and annotated them for a number of relevant features, including (a) the presence of a backchannel or brief token of agreement (e.g., *yeah*, *right*) in either the overlapped or overlapping utterance, (b) the presence of a period of silence within 200 ms from the beginning of the overlap period, (c) the presence of a transition relevance place (a point of syntactic, prosodic and pragmatic completion) in the overlapped turn within the 500 ms leading to the overlap, (d) an abandoned (i.e., syntactically and prosodically incomplete) utterance by any of the two speakers during or immediately following the overlap interval, and (e) the presence of repeated syllables or words in any of the two speaker’s utterances during or immediately following the overlap interval. Other recurrent features observed during or close to the overlap interval, such as laughter and disfluencies, were also annotated.

**Table 2** shows the most frequent features observed in the data (note that the features are not mutually exclusive). Interestingly, the majority of overlap cases (73%) involved a backchannel. Backchannels, especially continuers like “mm hm” or “uh huh,” are not construed as full turns, but rather pass up the opportunity to take a turn, and are thus principled intrusions into the other’s speech (Schegloff, 2000). It should be noted that, in half of the between-overlaps, it was not the backchannel that incurred the overlap, but rather the main speaker who produced an utterance in overlap with the backchannel. We also noted that overlapping backchannels often occurred after a TRP or a period of silence, suggesting that their timing is sensitive to specific cues in the main speaker’s turn (cf. Gravano and Hirschberg, 2009).

**Table 2:** Frequency of seven features in a subset of 200 cases of overlap (100 between-overlaps, and 100 within-overlaps) extracted from our Switchboard data.

	Between-overlaps (*n* = 100)	Within-overlaps (*n* = 100)	Percentage in total (*n* = 200)
Backchannel or agreement present	74	72	73%
Follows TRP (<500 ms)	23	51	37%
Follows silence (simultaneous start)	21	37	29%
Abandoned turn	21	18	19.5%
Follows disfluency in interlocutor’s turn	4	18	11%
Repeated syllables or words	4	12	8%
Any of the six features above	93	97	95%

Note that observations can exhibit more than one feature at the same time (e.g., cases of overlap after a period of silence involving a backchannel.

The second most common feature (37%) was the presence of a possible transition-relevance place (i.e., a point of syntactic, intonational, and pragmatic turn completion) in the overlapped turn within a time window of 500 ms before the start of the overlap. Another common feature was a period of silence (29%). In cases with this feature, one of the two speakers produced an utterance briefly after her interlocutor. These cases often involved a backchannel (*n* = 35, or 60%), or resulted in one of the two speakers abandoning their turn prematurely before reaching a point syntactic and prosodic completion (*n* = 14, or 24.1%). The presence of a disfluency in the utterance of the overlapped speaker before the start of the overlap (i.e., short silent pauses, repeated syllables or words, or noticeable decreases in speech rate) was also common. In these cases, it seems that the recipient produced a backchannel in response to the disfluency at a point when the interlocutor had already resumed her turn, causing overlap. In total, cases exhibiting one or more of these six features accounted for 95% of the data.

The remaining 10 cases involved three terminal between-overlaps affecting the last syllable of the previous turn, two cases exhibiting laughter by one of the speakers, two cases involving a turn-initial particle (i.e., *uhm* and *well*) produced in overlap with the last syllable of the preceding turn, one case with a speaker talking to someone else in the room, and one case of overlap due a clear phonetic segmentation error in the annotation.

Our analysis thus confirms that overlaps, though reasonably common (30% of transitions), are of short duration (i.e., less than 5% of the speech signal; between-overlaps have a modal duration 96 ms), occur largely in principled places (e.g., in between-overlaps, after possible completions, in simultaneous turn-starts), and mostly involve backchannels (which do not constitute full turns). In light of these observations, we conclude that the vast majority of instances of overlap in our dyadic conversations are consistent with the turn-taking system proposed by Sacks et al. (1974).

## 6. Psycholinguistics

Psycholinguistic processing puts tight constraints on any psychologically real model of turn-taking. Here we first draw attention to the early sensitivity to turn-taking in child development. Then we consider three main psycholinguistic aspects: predictive theories of language comprehension, studies of language production (from conceptual planning to speech articulation), and ideas about the relation between these two processes. Finally we turn to a small number of experimental studies aimed at understanding the relationship between comprehension and production processes in turn-taking.

### 6.1. ‘Proto-Conversation’ and Turn Taking in Human Development

Parallel to Sacks et al. (1974), in the 1970s there was an interest in children’s acquisition of turn-taking abilities. Trevarthen (1977) and Bruner (1983) coined the term “protoconversation” for the rhythmic alternation of vocalizations between care-giver and infant in the early months of life, and its systematic properties were demonstrated by Bateson (1975), with average turn transitions of about 1.5 s at 3 months. Subsequent work showed that this gap reduced in the following pre-linguistic months to around 800 ms (Jasnow and Feldstein, 1986; Beebe et al., 1988). Such early onset suggests that turn-taking may have an instinctive basis. Garvey and Berninger (1981) showed that the gap duration *increased* toward a second and a half in toddlers, presumably because of the cognitive difficulties of language production, and remained at around a second even for 5-year-olds [this slow convergence with adult norms has recently been confirmed for a larger sample by Stivers et al. (under review)].

After a long pause, there is now renewed interest in the development of turn-taking and its timing in children, and we now have better data, methods and concepts. Using audiovisual corpus techniques, Hilbrink et al. (submitted) have confirmed the general pattern earlier reported, namely relatively fast transitions in the prelinguistic period, with a slowing down as language starts to be comprehended at 9 months. Using eye-tracking of infants watching dyadic interaction, several studies have shown that 3-year-olds observers of dyadic conversations between two adults can *anticipate* speaker transitions (Tice and Henetz, 2011; Casillas and Frank, 2013, submitted; Keitel et al., 2013). Although the gaze shifts tend to occur in the gap (i.e., not in overlap with the turn preceding the floor transition), known saccade latencies for infants are c. 300 ms (Fernald et al., 2008), showing that they have often systematically detected the end of the turn before the gap. Researchers have also been able to show that by 3 year-olds, children are using intonation to do this projection of turn-ends (Keitel et al., 2013). Casillas and Frank (submitted) found that 3-year-olds were just as good at anticipating speaker change as adults, and did so more after questions than statements. They then looked at younger infants and filtered the speech, so they could distinguish whether prosody or lexico-syntax was enabling this anticipation. They found that 1 and 2 year-olds were better than chance at anticipating transitions, and that anticipation improves with age. Children under 3 were better in the prosody-only condition (with words filtered out) than they were in the words-only condition (with prosody filtered), indicating an early advantage for prosody (adults only showed an advantage for words + prosody). Clearly these studies confirm that projection is a real phenomenon, that it is learnt early, and that prosody plays an important role in this ability. They also indicate that turn-taking is established before language, that it forms a framework for language acquisition, and that the complexities of language slow down the framework through middle childhood.

### 6.2. Predictive Language Comprehension

Early in the history of psycholinguistics, Chomsky (1969, p. 57) insisted that probability and prediction had no possible role to play in a scientific theory of language: “It must be recognized that the notion ‘probability of a sentence’ is an entirely useless one, under any known interpretation of this term.” He reasoned that a grammar bounds a discrete infinity, and hence there was no core role for prediction in language understanding. The spell lasted decades, but meanwhile both engineering and psycholinguistic experiments have demonstrated a core role for statistical learning and estimation in language comprehension. For example, eye-movement studies in the visual world paradigm show that listeners predict upcoming entities from likely collocations (e.g., hearing “the boy is eating” participants look at the cake and not the ball in the picture). Determiners (e.g., French *un* vs. *une*), Adjectives (“freshly baked”) and verbs (“eat”) can predict nouns by their selectional restrictions; in language that have verbs at the end of the sentence like Japanese, participants can use the nouns to predict the verbs (Altmann and Kamide, 1999; Kamide et al., 2003). Another source of insight comes from EEG, where it can be shown that the syntactic frame is used to predict upcoming material. For example, when the sentential context leads one to expect a specific noun (‘she carried the eggs in a …’) but the gender of an incoming article is incongruous an N400 may be evoked before the noun itself is encountered (e.g., in Spanish *una canasta* ‘a basket’ vs. *un costal* ‘a sack’). These studies use the inverse correlation between the cloze probability and the amplitude of an N400 to demonstrate prediction (it is hard in fact to distinguish prediction from integration difficulties; see Kutas et al., 2011 for review). Predictive language comprehension is not only achieved on the basis of semantic and morphosyntactic regularities. In an experiment involving visual searches under the directions of a confederate, Ito and Speer (2008) showed that participants could anticipate referents on a screen (e.g., a “drum” vs. a “ball”) on the basis of the location of contrastive pitch accents in the vocal instructions being given to them (e.g., “now take the GREEN ball” vs. “now take the green BALL”). Listeners therefore appear to be able to use different sorts of linguistic information (i.e., semantic, morphosyntactic, prosodic) in order to predict the content of an incoming utterance. For an overview of recent work on predictive language understanding see Pickering and Garrod (2013).

Recent investigations have also shown direct connections of these predictive inferences to projection in conversation. Gisladottir et al. (2015) conducted an EEG experiment in which participants listened to mini-dialogs of two turns. The second turn (e.g., “I have a credit card”) could be invariant over three conditions, a question like “How are you going to pay?,” an offer like “I can lend you the money,” or a trouble announcement like “I don’t have any money.” In each of three contexts, the same second turn performs a different speech act (i.e., an answer, a declination, or an offer). The EEG signal, averaged over many such adjacency pairs, showed that very early (often in the first 400 ms) the different speech act forces of the response were predicted. Speech act detection is the precondition to response preparation, and it seems to be an early predictive process. A second relevant study (Magyari et al., 2014) looked at the EEG signal of participants listening to turns extracted from genuine conversations whose turn-endings they had to predict by pressing a button. These turns had already been sorted into unpredictable vs. predictable by a cloze test, where participants had to guess the missing words of items cut-off at various points. The predictable turns (compared to the unpredictable ones) showed a very early EEG signature of preparation to respond about half way through the turn (c. 1200 ms before the end). Recently Riest et al. (2015) show experimentally that responses based on prediction are not significantly different than those based on pre-knowledge. They also incidentally attempt to estimate stochastic tendencies for possible reactive responses (although these stimuli are non-linguistic and do not have the uncertainty associated, e.g., with voiceless stops). These studies together suggest that quite long-range prediction is normally involved in understanding language in a conversational mode.

### 6.3. Latencies in Language Production

There are striking differences between language comprehension and production despite the fact that the processes must be intimately related. One of the clearest differences is in processing speed. Speech production is a bottleneck on the whole language system: at about an average of seven syllables per second, speech can be estimated to have a bit-rate of under 100 bps (Levinson, 2000, p. 28). Studies of language production show that pre-articulation processes run three or four times faster than actual articulation (Wheeldon and Levelt, 1995). Studies of language comprehension under compression show that people can parse and comprehend speech at three or four times the speed of speech production (Calvert, 1986, p. 178; Mehler et al., 1993). Speech encoding is one part of the process that has to be strictly serial. Articulation is thus a severe bottleneck on communication, and the system compensates by utilizing pragmatic heuristics in production that augment the coded message (Levinson, 2000).

Happily, there have been extensive studies of language production that allow us to quantify the latency in each part of the production process, using picture naming as a task (Levelt, 1989). The average reaction from seeing a picture to beginning the naming of has been estimated at 600 ms (Indefrey and Levelt, 2004, p. 106). The literature unfortunately gives no ranges or standard deviations, with the exception of a study by Bates et al. (2003), which provides cross-linguistic averages that are much longer at over 1000 ms, with all minimums over 650 ms. Indefrey and Levelt (2004, p. 108), on the basis of a meta-study of available experiments, propose approximate figures for each stage of the process, which we show in **Table 3**.

**Table 3:** Estimated average time windows for successive operations in spoken word encoding (Indefrey and Levelt, 2004, p. 108).

Operation	Duration (ms)
Conceptual preparation (from picture onset to selecting the target concept)	175
Lemma retrieval Form encoding:	75
Phonological code retrieval	80
Syllabification	125
Phonetic encoding (till initiation of articulation)	145
Total	600

For multiword utterances, the effect is not linear. Naming two nouns takes 740–800 ms before output begins, with evidence that the processing of the second noun has begun but not finished by this time, while 900 ms is required for three word utterances (Schnur et al., 2006). Most of these studies incidentally (but not Bates et al., 2003) involve pre-familiarization of the words and pictures, so these response times are effectively after some amount of priming.

There is also good information on the planning required for sentence production from eye-movement studies. When participants are shown pictures of simple transitive or intransitive scenes (e.g., boy kicking ball, girl running), it takes about 1500 ms before speech output begins (Griffin and Bock, 2000; Gleitman et al., 2007). Interestingly, what happens within this 1500 ms is language-dependent – for example verb-first languages show rather different visual scanning of the pictures than verb medial languages (Norcliffe et al., 2015), but the latencies remain similar.

During this period of planning for language production, output processes involve the synergies between multiple speech organs. For example, breathing for speaking may need to be initiated. Earlier studies have shown that such breathing activity involves a number of latencies: first, c. 140–320 ms must be allowed for from the time the decision to inhale is made till the time the signal reaches the intercostal muscles (Draper et al., 1960); second, the inhalation time in spontaneous dialog is typically over 500 ms long (McFarland, 2001, p. 136). Together, these numbers suggest a latency of at least 500–800 ms prior to speech. In a recent study of breathing in conversation (Torreira et al., 2015, this volume), we have shown that short responses to questions are often made on residual lung air, whereas longer responses are likely to require a planned inhalation. The actual inhalation most typically starts briefly (i.e., 15 ms) after the end of the interlocutor’s question, and it is probably triggered just before the phonological retrieval process for the first word of the planned response. Thus the breathing data suggests that whether or not inhalation is required is a decision made during conceptual planning of the response, and that the trigger for inhalation, most typically produced during the last few hundred ms of the interlocutor’s turn, is often based on a prediction that the current speaker will imminently end her turn.

Recent studies of vocal preparation using ultrasound techniques show that tongue movements preceding speech production start considerably before the acoustic signal, with clear preparation between 120 and 180 ms prior to the acoustic release (Schaeﬄer et al., 2014) and with some effects detectable as early as 480 ms (Drake et al., 2014). Although not yet studied in a conversational context (although see de Vos et al., 2015, this volume, for the parallel in signed conversation), these measurements provide further estimates of the latencies involved in language production. These latencies are perhaps not surprising given the complexity of language encoding and the need for the processes to be funneled into a single, serial sequence of operations. Donders (1869) showed that reaction time varies with the number of choices that need to be made, and Hick’s Law (Hick, 1952) suggests this relation is generally logarithmic (reaction time will increase with decision time, where decision time T = log_2_(n) and n is the number of equally probable choices). When one considers that in production single words have to be plucked from a word lexicon consisting of over 20,000 entries, one can see immediately the processing problems involved. Combined with the relatively slow nature of nerve conduction (known since Helmholtz, 1850), and the complexity of the coordination of c. 100 muscles involved in articulation (Levelt, 1989), slow reaction times can be expected.

To summarize, language production involves latencies of well over half a second, and a multi-word utterance is likely to involve a second or more of processing before articulation begins. Although the conversational context may expedite some of these processes, the bulk of this latency is attributed to the phonological and phonetic encoding processes (as are frequency effects, Jescheniak and Levelt, 1994) which are probably not compressible.

### 6.4. Experimental Studies of Turn-Taking

There have been as yet relatively few experimental studies of turn-taking, due to the difficulties involved in gaining sufficient experimental control in free interaction. However, indirect light has been thrown on the mechanisms by extracting turns from conversation and experimentally testing when and how participants detect turn ends. De Ruiter et al. (2006) extracted turns from a corpus of conversations in Dutch, and got participants to press a button in anticipation of turn endings. They manipulated the turns so that there were versions where pitch information was filtered out (No Pitch), where the words were masked but the pitch preserved (No Words), where both were filtered (No Pitch, No Words) and finally where amplitude variation was also removed (Noise condition). They found that accuracy of turn-end anticipation was preserved under No Pitch, but significantly lost under No Words, and hugely affected under the other conditions, and they claim that “The conclusion is clear: lexicosyntactic structure is necessary (and possibly sufficient) for accurate end-of-turn projection, while intonational structure, perhaps surprisingly, is neither necessary nor sufficient” (De Ruiter et al., 2006, p. 531).

This study suggested then that lexicon and syntax are the key guide to turn-structure and completion. But there are aspects of prosody and articulation that may be critical, and in the normal case intonation may also be an important signal. To test this, Bögels and Torreira (in press) used turns taken from multiple scripted interviews, with questions like “So you’re a student at Radboud University?” (long version) vs. “So you’re a student?” (short version). The short versions exhibited a higher maximum pitch and greater duration on the last syllable of the word ‘student’ than the long versions, due to the presence of an intonational phrase boundary at the end of this word in the short questions, but not in the long questions. They cross-spliced their materials in different ways, and did the same button-press experiment as De Ruiter et al. (2006). Participants often false alarmed (pressed the button) at ‘student’ when a phrase-final word was cross-spliced into the middle of the long version – they were clearly using the prosodic information to anticipate turn closure. Participants were also presented with truncated long sentences ending in a syntactic point of completion, but lacking a final intonation phrase boundary: now participants only reacted on average around 400 ms after the end of the stimulus, suggesting that in this case participants’ button presses were produced in reaction to silence. On the other hand, in another condition consisting of similar words, but featuring a final intonational boundary, RTs were around 100 ms on average, suggesting reaction to or local prediction of an intonationally well-formed question end. It should be noted that while pitch had been filtered in the De Ruiter et al. (2006) study, duration and other phonetic cues to prosodic structure were still present in their filtered No Pitch condition. This new study shows that participants do use prosodic cues to judge turn-ending. What the de Ruiter et al. study does establish is that they need to be integrated with the lexical/syntactic information to carry turn-ending indications.

There are other experimental techniques that can be used to explore turn-taking. One is to use confederates (Bavelas and Gerwing, 2011), another to use the visual world paradigm with eye-tracking (Sjerps and Meyer, 2015). The latter study, using a dual task paradigm, found that maximal interference in the non-linguistic task occurred 500 ms before the end of the incoming turn (see also Boiteau et al., 2014); however, the linguistic task involved visual monitoring and was non-contingent with the incoming turn, so was far removed from conversation.

A method that combines control with live interaction involves alternating live and pre-recorded responses in such a way that participants are unaware of the manipulation (Bögels et al., 2014). In a recent study, we exploited this technique in a quiz-game (Bögels et al., submitted). Participants were recorded for EEG in a shielded room, and could not see the quiz master – this allowed some of the interaction to be live, some pre-recorded. The quiz questions were designed so that in some the answer was available early, and that in others the answer was available only toward the very end of the question, as in:

Which character, also called 007, appears in the famous movies? (Early)

*Which character from the famous movies, is also called 007? (Late)*.

In a second experiment, participants heard the same questions but did not have to answer them. Instead, they only had to remember them, as prompted by later probes. The neural patterns were then compared with those in the first experiment, where participants had to verbally respond, to the second where they only had to comprehend and memorize. The results revealed a clear neural signature associated with production, localized in the appropriate areas, occurring within 500 ms of the point at which a plausible answer to the question became available. Bögels and colleagues interpreted this as showing that participants begin planning their response as soon as they can, up to a second or more before the incoming turn ends.

### 6.5. The Core Psycholinguistic Puzzle

From a psycholinguistic point of view, turn-taking presents the following puzzle: in spite of the long latencies involved in language production (600–1500 ms or more), participants often manage to achieve smooth turn transitions (with the most typical gaps as little as 100–300). As a solution to this puzzle, we suggest that comprehension is predictive, even more so than is currently thought. As soon as possible, participants extract the speech act of the incoming utterance, which is the *sine qua non* for planning their appropriate response. In order to overcome the production latencies, they must also start the planning and encoding of the response as soon as possible.

This suggests that there is a significant overlap of comprehension and production processes. Given an average turn (approximated as an interpausal unit in our Switchboard Corpus data) of 1680 ms, somewhere in the middle response preparation may already be underway. This provides a second central puzzle: conversation involves constant double tasking, and this double tasking uses the same language system. The difficulty of the puzzle is increased when one takes into account the findings that both comprehension and production use much of the same neural circuitry (Segaert et al., 2011). It is plausible that the difficulty here is overcome through rapid task switching, and the gradual switch of resources from comprehension of the incoming turn toward production of the response.

Pickering and Garrod (2013) outline a general model of psycholinguistic processing, suggesting that production and comprehension are intimately intermeshed. Just as generally in action control, forward prediction of one’s own action is performed to correct deviations, so in interaction forward prediction of the other’s actions is used to check perception, and aid preparation of response. This is a nice account, but the complexities rapidly multiply. Listeners, on this account, are both using their full comprehension system, and running a fast simulation of the other’s production in order to predict the outcome. Now, given the turn-taking facts established above, we must add to this computational burden the need to simultaneously prepare one’s own turn in advance involving both the full production system and a hypothesized fast forward predictor. So the poor listener who is about to respond has not only the full comprehension and production processes running simultaneously, but also two fast prediction systems (one for self, one for other). This quadruple tasking looks unlikely, especially as similar tasks are hard to multitask. Additional problems are that unlike physical action prediction, which can be estimated by a few heuristics, it is not clear how a fast approximate language prediction system would be feasible especially in production – producers have to grind through the syntax to find, e.g., what order to put words in. More likely the real production system may be involved *minus* the phonological and phonetic encoding, which account for the bulk of the production latency.

In any case, regardless of how this is achieved, the experimental and corpus studies reviewed in this section converge in showing that participants in conversation often anticipate the content of the others’ turns well in advance, and that they use that information to prepare their response early.

## 7. Models of Turn-taking

Let us now gather together how the observations and inferences discussed above constrain viable models of turn-taking. Any adequate model must be consistent with a number of observations and constraints, as originally noted by Sacks et al. (1974, p. 700). We are now, however, able to add both additional constraints and a certain amount of temporal precision to those early observations:

(1) Turns are mostly short (mean 1680 ms, median 1227 ms; cf. see Section 5.2.1), consisting of one or more interjections, phrases or clauses at the syntactic level, and one or more intonational units at the prosodic level. Turn ends typically co-occur with points of both syntactic and prosodic completion.
(2) Intra-speaker gaps are longer by c. 150 ms than inter-speaker gaps (ten Bosch et al., 2005), suggesting ordered rules (the rights to the next turn unit belong first to the next speaker, and only if not exercised, to the current speaker).
(3) Inter-speaker gaps are most typically short, with modal values for FTOs falling between 100 and 200 ms (cf. **Figure 2**). Medium gaps and short overlaps are also common, although less so than short gaps.
(4) Lengthy gaps (over 700 ms) may carry semiotic significance (mostly, of an undesired or unexpected response; Kendrick and Torreira, 2015), thus contributing to propel fast timing.
(5) Overlaps, though common, are brief (with a mean of 275 ms at turn-transitions, and occupying less than 5% of the spoken signal in our telephone calls data). Overlaps are more common at turn transitions than within turns, and mostly involve back-channels, simultaneous first-starts, disfluencies, and other features predicted by Sacks et al. (1974).
(6) Turn-taking is established early in infancy, long before full linguistic competence, which actually appears to slow down response times; adult conversation timing is not achieved till late in middle childhood.
(7) Given the latencies of speech production (over 600 ms), incoming turns have to be predicted if accurate timing is to be achieved. EEG recordings suggest the production process in responsive turns starts as soon as the gist of the incoming turn can be detected.
(8) Turn-final cues seem to be used to recognize that a turn is definitely coming to an end. These cues are typically prosodic (e.g., phrase-final syllable lengthening and specific melodic patterns in many intonational languages) but also syntactic (e.g., syntactic closure), and in principle could be of other types too (e.g., gestural). In the appropriate pragmatic context, these turn-final cues can trigger the decision of the next speaker to articulate. From the point of view of social interaction, it is effective articulation that constitutes a point of no return (as opposed to other preparatory events preceding speech, such as pre-utterance inhalations and mouth noises).

### 7.1. The Standard Model and Alternatives

We have outlined above the Sacks et al. (1974) model of turn-taking as an opportunity-based or sharing system, regulated by normative rules. The behavioral patterns on this account are the outcome of joint, coordinated determination of turns, against a background of an assumption of rights to minimal turns. Not all turns are minimal of course, but in this case a bid must be made for an extended turn, as in:

(9) Terasaki, 1976, p. 53
D: I forgot to tell you the two best things that happen’ to me today.
R: Oh super=What were they?
D: I got a B+ on my math test ((material omitted)) and I got an athletic award.

An alternative model is the turn-end signaling system proposed by Duncan (1972), also mentioned above, under which the system is wholly in the control of the current speaker, who has exclusive rights and signals transfer at the end of the turn. In contrast, Sacks et al. (1974) held that “It is misconceived to treat turns as units characterized by a division of labor in which the speaker determines units and boundaries,” instead, “the turn as a unit is interactively determined.”

Duncan (1972, p. 286) proposed a simple rule of the sort “The auditor may take his speaking turn when the speaker gives a turn-yielding signal.” Such a system would be in effect like the “over and out” cuing at the end of turns on a two-way (half duplex) radio which permits hearing or talking but not both at once by a single party. Such a system predicts that overlap can only occur when “over” cues are mistakenly given or overridden; the large incidence of overlaps in corpora, and their clustering at principled locations (like overlapped tags or address forms) is then hard to reconcile with such a model. As mentioned, the model presumed that these turn-yielding signals such as intonation are context-independent, but in fact we know they are not – e.g., in English final rising intonation in a question may signal finality but in a statement continuation; thus their interpretation would have to be embedded in complex comprehension processes. The model is in any case very partial: it tells us nothing about how or why people should initiate a turn, why turns are generally short, how multiple participants can be integrated into a single conversation, how overlap is resolved, and so forth. But it may add a component to a more complex overall model.

### 7.2. Toward an Adequate Psycholinguistic Model of Turn Taking – Cognitive Processes in the Responder^1^

We believe that the property list in Section 7 above puts fairly narrow constraints on a possible model of turn-taking. One area of particular interest is the temporal constraints that turn-taking imposes on language processing, given that conversational interchange is the core form of language use. These constraints are funneled into one crucial link in the system, namely, the current addressee preparing to respond. Here we consider the cognitive processes that must be involved.

The crucial questions concern what factors govern the decision making process that lies behind the initiation and timing of response. While turn-final cues in the incoming turn seem likely to play a role, they cannot be sufficient given the long latencies in language planning and production. To overcome these long latencies, predictive comprehension must be involved, together with a strategy of early beginnings to production. Bögels et al. (submitted) suggest that production begins as soon as it can – that is, as soon as the speech act content of the incoming turn is clear. This implies of course dual-tasking, perhaps by rapid alternation (‘time sharing’). A new study using a dual-task paradigm and eye-tracking suggests that the heaviest interference is rather late (Sjerps and Meyer, 2015), and tied to looking-for-speaking which was postponed in this task toward the end of the incoming turn. Both early and late processes are almost certainly involved, but what exactly is happening, and when during natural conversation remains to be determined.

**FIGURE 3:**
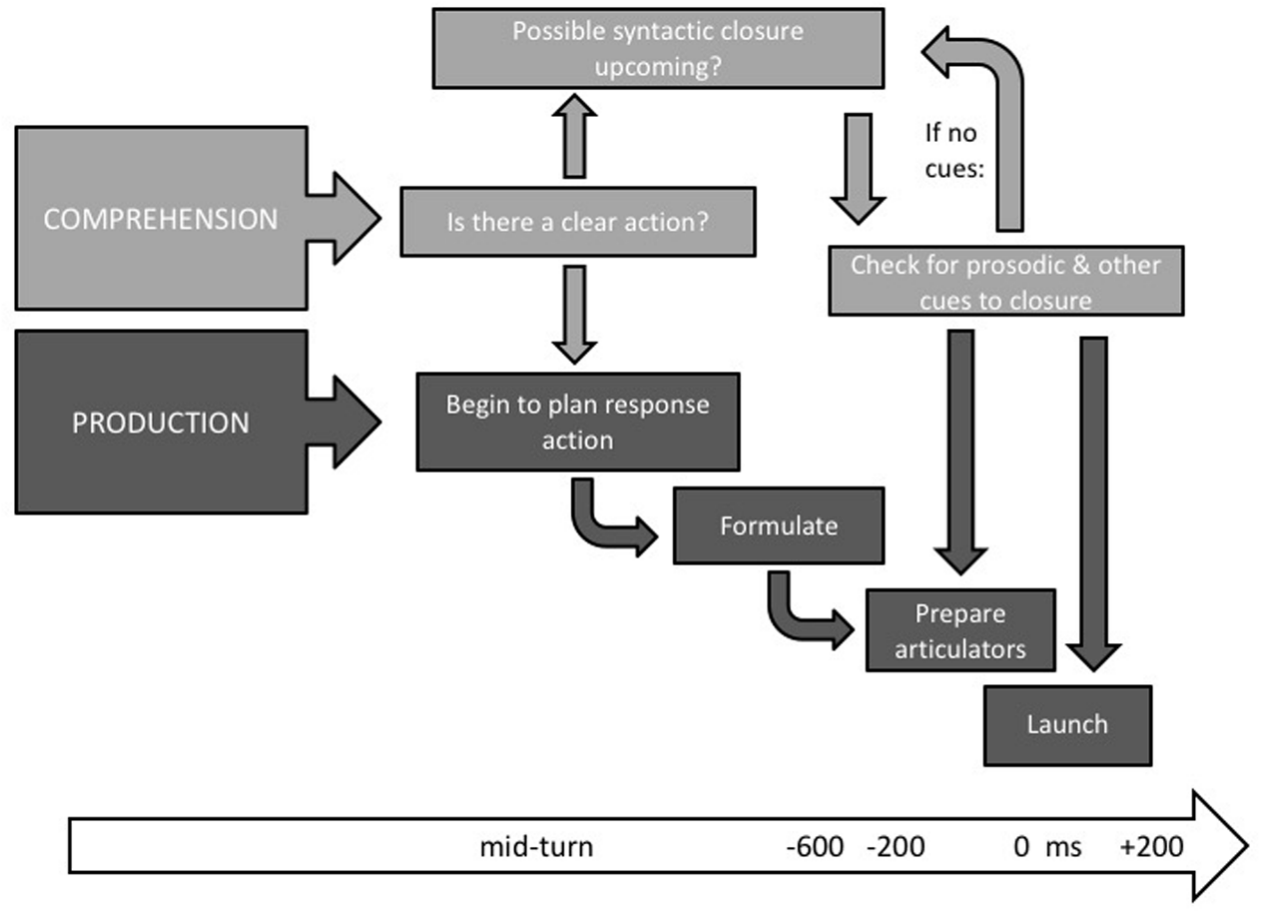
**Sketch of the interleaving of comprehension and production in the recipient of an incoming turn**.

The flowchart diagram in **Figure 3** sketches the cognitive processes that must minimally be at work in the recipient of a typical turn at talk during conversation. Predictive comprehension is underway early, and already by half way through more predictable turns will suggest a temporal envelope for completion (Magyari et al., 2014). If so, morphosyntax may provide most of the early clues to the overall structural envelope (e.g., turns beginning with *if* or *either* or *whenever* projects a two clause structure), so offering some long distance projection. Within the last half second or so, the actual words will often be predicted (Magyari and de Ruiter, 2012), and, within that same late time-frame, cues to imminent turn closure, usually prosodic and phonetic, are likely to appear (Local and Walker, 2012; Bögels and Torreira, in press), indicating a likely turn end.

A recipient’s first task is to identify or predict the speech act or action being carried out – both the illocutionary force and the likely propositional content. In cases in which the illocutionary force of the incoming utterance makes a floor exchange relevant or due, production planning may begin as soon as it is recognized, as suggested by the results in Bögels et al. (submitted). Production is, at least in the latter stages, serial, and proceeds through conceptualization, lemma retrieval, phonological retrieval, and phonetic encoding, following a time course that seems well understood (Indefrey, 2011), extending 600–1200 ms or more before articulation depending on the ease of retrieval and the length of the turn. In this model, early preparation is assumed, but actual articulation is held till turn-final cues (e.g., upcoming syntactic closure, a non-turn-keeping intonational phrase boundary) are detected, whereupon actual articulation is launched. Assuming these cues fall in the last half-second of the incoming turn, reaction to those will be sufficient to launch pre-prepared material so that it appears soon after the other’s turn is completed.

**Figure 3** sketches the kind of interaction between comprehension and production processes that must be involved in a typical turn transition (i.e., involving a FTO of c. 200 ms). There is an early gist comprehension with speech act apprehension sent as soon as possible to the production conceptualizer (see Levinson, 2013; Gisladottir et al., 2015). The production system may automatically begin to formulate right down to the phonology (Bögels et al., submitted), but with the actual articulation held in a buffer until the comprehension system signals an imminent completion of the incoming turn. Prior to that signal, it is likely that pre-articulation preparation (requiring c. 200 ms) of the vocal apparatus would be underway – this would include readying the vocal tract for the gestures to be made (see Drake et al., 2014; Schaeﬄer et al., 2014), and the decision to inhale prior to delivery of longer responses (Torreira et al., 2015, this volume).

Meanwhile the comprehension system continues to check the incoming signal for possible closure at both the syntactic and prosodic level. As soon as there are consistent signals of linguistic completion, a go-signal is sent to production, and any buffered articulation released. It is likely that visual monitoring of gesture can also be utilized for the go-signal (Duncan, 1974), but this awaits experimental confirmation.

This model is responsive to all the constraints listed in Section 7. What this model crucially adds is:

(a) an account of how responders can often respond with short latencies despite the long latencies of the production system;
(b) why the corpus statistical results reliably show a modal response with positive offsets of around 100–300 ms, reflecting the reaction time to the turn-final prosodic cues in the incoming turn (i.e., reaction to the go-signal, as hypothesized by Heldner and Edlund, 2010).

The model sketch in **Figure 3** is based on average, modal, and minimal temporal latencies reported in the literature. We would like to propose that this model is generally valid in the most frequent scenarios. If speakers launched their responses as early as they could without waiting for turn-final cues, we should expect overlapping or no-gap–no-overlap transitions to be the most common, rather than a short gap. And, if speakers typically launched language planning only after identifying turn-final cues, we should expect the most frequent transition times to involve at least half a second or more rather than short gaps of 100–300 ms. The model therefore captures the most typical turn transition values observed in conversational corpora.

What, however, accounts for the significant number of overlap and long gap cases observable in any conversation? A reviewer suggests that human factors such as lack of attention, pre-formulated agendas, and apparent involvement with actual minimal responsiveness may all be involved, and notes that apparent good timing may be achieved with buffers like particles. However, the evidence is that conversation is generally more demanding than that – for example 95% of questions get answers (Stivers, 2010), and particles like *well* and *uhm* in English are semiotically loaded and thus not empty buffers (Kendrick and Torreira, 2015), while Roberts et al. (2015) failed to find statistical differences in the timing of turns with and without such particles. In addition, it is likely that speakers sometimes use other turn-taking than the one sketched in **Figure 3**. For example, under competition for the floor, or when responding to highly predictable utterances, speakers may decide to launch articulation without waiting to identify turn-final cues. In cases of long transition latencies, speakers may not have been able to plan the initial stages of their turn early enough to launch articulation when the interlocutor’s turn-final cues become available. This may indeed be due to a low attentional level on the part of the speaker, or to the interlocutor’s turn being unclear in purpose until its end or simply to the complexity of the response required (Torreira et al., 2015, this volume).

## 8. Conclusion

This overview of work on turn-taking behavior over the last half century shows that turn-taking is a remarkable phenomenon, for it combines high temporal coordination between participants with the remarkable complexity and open-endedness of the language that fills the turns. The tension between these two properties is reflected in the development of turn-taking in childhood (Proto-Conversation’ and Turn Taking in Human Development), and it poses a substantial puzzle for psycholinguistic models (i.e., dual-tasking comprehension and production processes), which until recently have completely ignored this, the most central form of language use.

## Conflict of Interest Statement

The authors declare that the research was conducted in the absence of any commercial or financial relationships that could be construed as a potential conflict of interest.

## Footnotes

1 he ideas presented in this section were developed in collaboration with Mathias Barthel, Sara Bögels, and the other members of the INTERACT project at the Max Planck Institute for Psycholinguistics. See also Section 5.3 in Heldner and Edlund (2010) for a parallel proposal.

## References

[B1] Altmann G. Kamide Y. (1999). Incremental interpretation at verbs: restricting the domain of subsequent reference. *Cognition* 73 247–264. 10.1016/S0010-0277(99)00059-1 10585516

[B2] Bates E. D’Amico S. Jacobsen T. Székely A. Andonova E. Devescovi A. (2003). Timed picture naming in seven languages. *Psychon. Bull. Rev.* 10 344–380. 10.3758/BF03196494 12921412 PMC3392189

[B3] Bateson M. C. (1975). Mother-infant exchanges: the epigenesis of conversational interaction. *Ann. N. Y. Acad. Sci.* 263 101–113. 10.1111/j.1749-6632.1975.tb41575.x 1060428

[B4] Bavelas J. B. Gerwing J. (2011). The listener as addressee in face-to-face dialogue. *Int. J. Listening* 25 178–198. 10.1080/10904018.2010.508675

[B5] Beebe B. Alson D. Jaffe J. Feldstein S. Crown C. (1988). Vocal congruence in mother-infant play. *J. Psychol. Res.* 17 245–259. 10.1007/BF01686358 3411533

[B6] Bögels S. Barr D. Garrod S. Kessler K. (2014). Conversational interaction in the scanner: mentalizing during language processing as revealed by MEG. *Cereb. Cortex* 10.1093/cercor/bhu116 [Epub ahead of print]. PMC4537451 24904076

[B8] Bögels S. Torreira F. (in press). Listeners use intonational phrase boundaries to project turn ends in spoken interaction. *J. Phonet*.

[B9] Boiteau T. W. Malone P. S. Peters S. A. Almor A. (2014). Interference between conversation and a concurrent visuomotor task. *J. Exp. Psychol. Gen.* 143 295–311. 10.1037/a0031858 23421443 PMC3720820

[B10] Brady P. T. (1968). A statistical analysis of on-off patterns in 16 conversations. *Bell Sys. Tech. J.* 47 73–91. 10.1002/j.1538-7305.1968.tb00031.x

[B11] Bruner J. (1983). *Child’s Talk*. New York, NY: Norton.

[B12] Buck J. Buck E. (1976). ‘Synchronous fireflies’. *Sci. Am.* 234 74–85. 10.1038/scientificamerican0576-74 1273569

[B13] Byrd D. (1993). 54,000 American stops. *UCLA Work. Papers Phon.* 83 97–116.

[B14] Calhoun S. Carletta J. Brenier J. M. (2010). The NXT-format switchboard corpus: a rich resource for investigating the syntax, semantics, pragmatics and prosody of dialogue. *Lang. Resour. Eval.* 44 387–419. 10.1007/s10579-010-9120-1

[B15] Calvert D. R. (1986). *Descriptive Phonetics* 2nd Edn New York, NY: Thieme Medical Publisher.

[B16] Casillas M. (2014). “Taking the floor on time: delay and deferral in children’s turn taking,” in *Language in Interaction: Studies in Honor of Eve V. Clark* eds Arnon I. Casillas M. Kurumada C. Estigarribia B. (Amsterdam: Benjamins) 101–114. 10.1075/tilar.12.09cas

[B17] Casillas M. Frank M. C. (2013). “The development of predictive processes in children’s discourse understanding,” in *Proceedings of the 35th Annual Meeting of the Cognitive Science Society* eds Knauff M. Pauen M. Sebanz N. Wachsmuth I. (Austin, TX: Cognitive Society) 299–304.

[B19] Clayman S. (2013). “Turn-constructional units and the transition-relevance place,” in *Handbook of Conversation Analysis* eds Stivers T. Sidnell J. (Chichester: Wiley-Blackwell) 151–166.

[B20] Chomsky N. (1969). “Quine’s Empirical Assumptions,” in *Words and Objections* eds Davidson D. Hintikka J. (Dordrecht: Reidel) 53–68. 10.1007/978-94-010-1709-1_5

[B21] Couper-Kuhlen E. (2009). “Relatedness and timing in talk-in-interaction,” in *Where Prosody Meets Pragmatics* eds Barth-Weingarten D. Dehé N. Wichmann A. (Leiden: Brill) 257–276. 10.1163/9789004253223_012

[B22] Crystal T. House A. (1988). Segmental durations in connected-speech signals: current results. *J. Acoust. Soc. Am.* 83 1553–1573. 10.1121/1.395911 7130529

[B23] De Ruiter J. P. Mitterer H. Enfield N. J. (2006). Projecting the end of a speaker’s turn: a cognitive cornerstone of conversation. *Language* 82 515–535. 10.1353/lan.2006.0130

[B24] de Vos C. Torreira F. Levinson S. C. (2015). Turn-timing in signed conversations: coordinating stroke-to-stroke turn boundaries. *Front. Psychol.* 6:268 10.3389/fpsyg.2015.00268 PMC4371657 25852593

[B25] Donders F. C. (1869). “On the speed of mental processes,” in *Attention & Performance II* ed. and trans. Koster W. G. (Amsterdam: North-Holland) 412–431.

[B26] Drake E. Schaeﬄer S. Corley M. (2014). “Articulatory effects of prediction during comprehension: an ultrasound tongue imaging approach,” in *Proceedings of the 10th International Seminar on Speech Production* Cologne.

[B27] Draper M. H. Ladefoged P. Whitteridge D. (1960). Expiratory pressures and air flow during speech. *Br. Med. J.* 1 1837–1843. 10.1136/bmj.1.5189.1837 13818006 PMC1967952

[B28] Drew P. (2013). “Turn Design,” in *Handbook of Conversation Analysis* eds Stivers T. Sidnell J. (Chichester: Wiley-Blackwell) 131–149.

[B29] Duncan S. D. (1972). Some signals and rules for taking speaking turns in conversation. *J. Pers. Soc. Psychol.* 23 283–292. 10.1037/h0033031

[B30] Duncan S. D. (1974). On the structure of speaker-auditor interaction during speaking turns. *Lang. Soc.* 2 161–180. 10.1017/S0047404500004322

[B31] Fernald A. Zangl R. Portillo A. L. Marchman V. A. (2008). “Looking while listening: using eye movements to monitor spoken language comprehension by infants and young children,” in *Developmental Psycholinguistics: On-line Methods in Children’s Language Processing* eds Sekerina I. A. Fernandez E. M. Clahsen H. (Amsterdam: Benjamins) 97–135. 10.1075/lald.44.06fer

[B32] Ford C. E. Thompson S. A. (1996). “Interactional units in conversation: syntactic, intonational, and pragmatic resources for the projection of turn completion,” in *Interaction and Grammar* eds Ochs E. Schegloff E. A. Thompson S. A. (Cambridge: Cambridge University Press) 135–184.

[B33] Fry D. B. (1975). Simple reaction-times to speech and non-speech stimuli. *Cortex* 11 355–360. 10.1016/S0010-9452(75)80027-X 1222579

[B34] Garvey C. Berninger G. (1981). Timing and turn-taking in children’s conversations. *Discourse Process.* 4 27–57. 10.1080/01638538109544505

[B35] Gisladottir R. Chwilla D. Levinson S. C. (2015). Conversation electrified: ERP correlates of speech act recognition in underspecified utterances. *PLoS ONE* 10:e0120068 10.1371/journal.pone.0120068 PMC4368040 25793289

[B36] Gleitman L. R. January D. Nappa R. Trueswell J. C. (2007). On the give and take between event apprehension and utterance formulation. *J. Mem. Lang.* 57 544–596. 10.1016/j.jml.2007.01.007 18978929 PMC2151743

[B37] Godfrey J. Holliman E. McDaniel J. (1992). “SWITCHBOARD: telephone speech corpus for research and development,” in *Proceedings of the IEEE International Conference on Acoustics, Speech and Signal Processing (ICASSP)* (San Francisco, CA: IEEE) 517–520. 10.1109/icassp.1992.225858

[B38] Goodwin C. (1980). Restarts, pauses, and the achievement of mutual gaze at turn-beginning. *Soc. Inq.* 50 272–302. 10.1111/j.1475-682X.1980.tb00023.x

[B39] Gravano A. Hirschberg J. (2009). “Backchannel-inviting cues in task-oriented dialogue,” in *Proceedings of SigDial 2009* London 253–261.

[B40] Griffin Z. M. Bock K. (2000). What the eyes say about speaking. *Psychol. Sci.* 4 274–279. 10.1111/1467-9280.00255 11273384 PMC5536117

[B41] Hayashi M. (2013). “Turn allocation and turn sharing,” in *Handbook of Conversation Analysis* eds Stivers T. Sidnell J. (Chichester: Wiley-Blackwell) 167–190.

[B42] Heldner M. (2011). Detection thresholds for gaps, overlaps and no-gap-no-overlaps. *J. Acoust. Soc. Am.* 130 508–513. 10.1121/1.3598457 21786916

[B43] Heldner M. Edlund J. (2010). Pauses, gaps and overlaps in conversations. *J. Phon.* 38 555–568. 10.1016/j.wocn.2010.08.002

[B44] Helmholtz H. (1850). “Vorläufiger Bericht Über die Fortpflanzungs-Geschwindigkeit der Nervenreizung,” in *Archiv für Anatomie, Physiologie und wissenschaftliche Medicin* (Berlin: Veit & Comp.) 71–73.

[B45] Hick W. E. (1952). On the rate of gain of information. *Q. J. Exp. Psychol.* 4 11–26. 10.1080/17470215208416600

[B47] Indefrey P. (2011). The spatial and temporal signatures of word production components: a critical update. *Front. Psychol.* 2:255 10.3389/fpsyg.2011.00255 PMC3191502 22016740

[B48] Indefrey P. Levelt W. J. M. (2004). The spatial and temporal signatures of word production components. *Cognition* 92 101–144. 10.1016/j.cognition.2002.06.001 15037128

[B49] Ito K. Speer S. R. (2008). Anticipatory effects of intonation: eye movements during instructed visual search. *J. Mem. Lang.* 58 541–573. 10.1016/j.jml.2007.06.013 19190719 PMC2361389

[B50] Izdebski K. Shipp T. (1978). Minimal reaction times for phonatory initiation. *J. Speech Hear. Res.* 21 638–651. 10.1044/jshr.2104.638 745366

[B51] Jasnow M. Feldstein S. (1986). Adult-like temporal characteristics of mother-infant vocal interactions. *Child Dev.* 57 754–761. 10.2307/1130352 3720401

[B52] Jefferson G. (1984). “Notes on some orderliness of overlap onset,” in *Discourse Analysis and Natural Rhetoric* eds D’Urso V. Leonardi P. (Padua: Cleup Editore) 11–38.

[B53] Jefferson G. (1986). Notes on ‘latency’ in overlap onset. *Hum. Stud.* 9 153–183. 10.1007/BF00148125

[B54] Jescheniak J. D. Levelt W. J. M. (1994). Word frequency effects in speech production: retrieval of syntactic information and of phonological form. *J. Exp. Psychol. Learn. Mem. Cogn.* 20 824–843. 10.1037/0278-7393.20.4.824

[B55] Kamide Y. Altmann G. T. M. Haywood S. L. (2003). The time-course of prediction in incremental sentence processing: evidence from anticipatory eye movements. *J. Mem. Lang.* 49 133–156. 10.1016/S0749-596X(03)00023-8

[B56] Keitel A. Prinz W. Friederici A. D. von Hofsten C. Daum M. M. (2013). Perception of conversations: the importance of semantics and intonation in children’s development. *J. Exp. Child Psychol.* 116 264–277. 10.1016/j.jecp.2013.06.005 23876388

[B57] Kendon A. (1967). Some functions of gaze-direction in social interaction. *Acta Psychol.* 26 22–63. 10.1016/0001-6918(67)90005-4 6043092

[B58] Kendrick K. Torreira F. (2015). The timing and construction of preference: a quantitative study. *Discourse Process.* 52 255–289. 10.1080/0163853X.2014.955997

[B59] Kutas M. DeLong K. A. Smith N. J. (2011). “A look around at what lies ahead: prediction and predictability in language processing,” in *Predictions in the Brain: Using our Past to Generate a Future* ed. Bar M. (Oxford: Oxford University Press) 190–207.

[B60] Lerner G. H. (1991). On the syntax of sentences in progress. *Lang. Soc.* 20 441–458. 10.1017/S0047404500016572

[B61] Lerner G. H. (2002). “Turn-sharing: the choral co-production of talk-in-interaction,” in *The Language of Turn and Sequence* eds Ford C. Fox B. Thompson S. (Oxford: Oxford University Press) 225–256.

[B62] Levelt W. J. M. (1989). *Speaking: From Intention to Articulation.* Cambridge, MA: MIT Press.

[B63] Levinson S. (1983). *Pragmatics.* Cambridge: Cambridge University Press.

[B64] Levinson S. (2000). *Presumptive Meanings.* Cambridge, MA: MIT Press.

[B65] Levinson S. (2013). Recursion in pragmatics. *Language* 89 149–162. 10.1353/lan.2013.0005

[B66] Local J. Walker G. (2012). How phonetic features project more talk. *J. Int. Phon. Assoc.* 42 255–280. 10.1017/S0025100312000187

[B67] Magyari L. Bastiaansen M. C. M. De Ruiter J. P. Levinson S. C. (2014). Early anticipation lies behind the speed of response in conversation. *J. Cogn. Neurosci.* 26 2530–2539. 10.1162/jocn_a_00673 24893743

[B68] Magyari L. de Ruiter J. P. (2012). Prediction of turn-ends based on anticipation of upcoming words. *Front. Psychol.* 3:376 10.3389/fpsyg.2012.00376 PMC3483054 23112776

[B69] McFarland D. H. (2001). Respiratory markers of conversational interaction. *J. Speech Lang. Hear Res.* 44 128–143. 10.1044/1092-4388(2001/012) 11218097

[B70] Mehl M. R. Vazire S. Ramírez-Esparza N. Slatcher R. B. Pennebaker J. W. (2007). Are women really more talkative than men? *Science* 317 82 10.1126/science.1139940 17615349

[B71] Mehler J. Sebastian N. Altmann G. Christophe A. Pallier C. (1993). Understanding compressed sentences: the role of rhythm and meaning. Paper presented at the Temporal information processing in the nervous system. *Ann. N. Y. Acad. Sci.* 682 272–282. 10.1111/j.1749-6632.1993.tb22975.x 8323119

[B72] Munhall K. Gribble P. Sacco L. Ward M. (1996). Temporal constraints on the McGurk effect. *Percept. Psychophys.* 58 351–362. 10.3758/BF03206811 8935896

[B73] Norcliffe E. Konopka A. Brown P. Levinson S. C. (2015). Word order affects the time-course of sentence formulation in Tzeltal. *Lang. Cogn. Neurosci.* 10.1080/23273798.2015.1006238

[B74] Norwine A. C. Murphy O. J. (1938). Characteristic time intervals in telephonic conversation. *Bell Syst. Tech. J.* 17 281–291. 10.1002/j.1538-7305.1938.tb00432.x

[B75] Pickering M. J. Garrod S. (2013). An integrated theory of language production and comprehension. *Behav. Brain Sci.* 36 329–347. 10.1017/S0140525X12001495 23789620

[B76] Pomerantz A. Heritage J. (2013). “Preference,” in *Handbook of Conversation Analysis* eds Stivers T. Sidnell J. (Chichester: Wiley-Blackwell) 210–228.

[B77] Riest C. Jorschick A. B. De Ruiter J. P. (2015). Anticipation in turn-taking: mechanisms and information sources. *Front. Psychol.* 6:89 10.3389/fpsyg.2015.00089 PMC4313610 25699004

[B78] Roberts F. Margutti P. Takano S. (2011). Judgments concerning the valence of inter-turn silence across speakers of American English, Italian, and Japanese. *Discourse Process.* 48 331–354. 10.1080/0163853X.2011.558002

[B79] Roberts S. G. Torreira F. Levinson S. C. (2015). The effects of processing and sequence organization on the timing of turn taking: a corpus study. *Front. Psychol.* 6:509 10.3389/fpsyg.2015.00509 PMC4429583 26029125

[B80] Rossano F. (2013). “Gaze in conversation,” in *Handbook of Conversation Analysis* eds Stivers T. Sidnell J. (Chichester: Wiley-Blackwell) 308–329.

[B81] Sacks H. Schegloff E. Jefferson G. (1974). A simplest systematics for the organization of turn-taking in conversation. *Language* 50 696–735. 10.1353/lan.1974.0010

[B82] Schaeﬄer S. Scobbie J. M. Schaeﬄer F. (2014). “Measuring reaction times: vocalisation vs. *articulation*,” in *Proceedings of the 10th International Seminar on Speech Production* Cologne.

[B83] Schegloff E. A. (2000). Overlapping talk and the organization of turn-taking for conversation. *Lang. Soc.* 29 1–63. 10.1017/S0047404500001019

[B84] Schnur T. T. Costa A. Caramazza A. (2006). Planning at the phonological level during sentence production. *J. Psycholinguist. Res.* 35 189–213. 10.1007/s10936-005-9011-6 16502144

[B85] Sebanz N. Knoblich G. K. (2008). “From mirroring to joint action,” in *Embodied Communication*, eds Wachsmuth I. Lenzen M. Knoblich G. K. (Oxford: Oxford University Press) 129–150.

[B86] Segaert K. Menenti L. Weber K. Hagoort P. (2011). A paradox of syntactic priming: why response tendencies show priming for passives, and response latencies show priming for actives. *PLoS ONE* 6:e24209 10.1371/journal.pone.0024209 PMC3191135 22022352

[B87] Sellen A. J. (1995). Remote conversations: the effects of mediating talk with technology. *Hum. Comput. Interact.* 10 401–444. 10.1207/s15327051hci1004_2

[B88] Shipp T. Izdebski K. Morrissey P. (1984). Physiologic stages of vocal reaction time. *J. Speech Hear. Res.* 27 173–178. 10.1044/jshr.2702.173 6330455

[B89] Sjerps M. Meyer A. (2015). Variation in dual-task performance reveals late initiation of speech planning in turn-taking. *Cognition* 136 304–324. 10.1016/j.cognition.2014.10.008 25522192

[B90] Stivers T. (2010). An overview of the question-response system in American English conversation. *J. Pragmatics* 42 2772–2781. 10.1016/j.pragma.2010.04.011

[B91] Stivers T. Enfield N. J. Brown P. Englert C. Hayashi M. Heinemann T. (2009). Universals and cultural variation in turn-taking in conversation. *Proc. Natl. Acad. Sci. U.S.A.* 106 10587–10592. 10.1073/pnas.0903616106 19553212 PMC2705608

[B92] Stivers T. Enfield N. J. Levinson S. C. (2010). Question-response sequences in conversation across ten languages: an introduction. *J. Pragmatics* 42 2615–2619. 10.1016/j.pragma.2010.04.001

[B94] ten Bosch L. Oostdijk N. Boves L. (2005). On temporal aspects of turn-taking in conversational dialogues. *Speech Commun.* 47 80–86. 10.1016/j.specom.2005.05.009

[B96] ten Bosch L. Oostdijk N. de Ruiter J. P. (2004). “Turn-taking in social talk dialogues: temporal, formal, and functional aspects,” in *Proceedings of the Ninth Conference on Speech and Computer (SPECOM 2004)* Saint-Petersburg: St.Petersburg 454–461.

[B97] Terasaki A. (1976). *Pre-announcement Sequences in Conversation (No. 99)*. Irvine, CA: University of Irvine, Social Sciences.

[B98] Tice M. Henetz T. (2011). “Turn-boundary projection: looking ahead,” in *Proceedings of the 33rd Annual Conference of the Cognitive Science Society*, eds Carlson L. Hölscher C. Shipley T. (Austin, TX: Cognitive Science Society) 838–843.

[B99] Torreira F. Bögels S. Levinson S. C. (2015). Breathing for answering: the time course of response planning in conversation. *Front. Psychol.* 6:284 10.3389/fpsyg.2015.00284 PMC4357202 25814976

[B100] Trevarthen C. (1977). “Descriptive analyses of infant communicative behaviour,” in *Studies in Mother-Infant Interaction* ed. Schaffer H. R. (London: Academic Press) 89–117.

[B101] Walker G. (2013). “Phonetics and prosody in conversation,” in *Handbook of Conversation Analysis* eds Stivers T. Sidnell J. (Chichester: Wiley-Blackwell) 455–474.

[B102] Weilhammer K. Rabold S. (2003). “Durational aspects in turn taking,” in *Proceedings of the International Conference of Phonetic Sciences* Barcelona.

[B103] Wells B. Macfarlane S. (1998). Prosody as an interactional resource: turn-projection and overlap. *Lang. Speech* 41 265–294. 10.1177/002383099804100403 10746359

[B104] Wheeldon L. R. Levelt W. J. M. (1995). Monitoring the time-course of phonological encoding. *J. Mem. Lang.* 34 311–334. 10.1006/jmla.1995.1014

[B105] Wilson M. Wilson T. P. (2005). An oscillator model of the timing of turn-taking. *Psychon. Bull. Rev.* 12 957–968. 10.3758/BF03206432 16615316

[B106] Wilson T. P. Zimmerman D. H. (1986). The structure of silence between turns in two-party conversation. *Discourse Process.* 9 375–390. 10.1080/01638538609544649

[B107] Yngve V. H. (1970). On getting a word in edgewise. *Papers from the Sixth Regional Meeting of the Chicago Linguistic Society.* Chicago: Chicago Linguistic Society.

